# Diagnostic pitfalls: intramyocardial lymphoma metastasis mimics acute coronary syndrome in a diffuse large B cell lymphoma patient—case report

**DOI:** 10.1186/s12245-021-00352-x

**Published:** 2021-05-07

**Authors:** Lilla Prenek, Klára Csupor, Péter Beszterczán, Krisztina Boros, Erika Kardos, András Vorobcsuk, Miklós Egyed, Ádám Kellner, Péter Rajnics, Csaba Varga

**Affiliations:** 1 Department of Emergency Medicine, Teaching Hospital Mór Kaposi, Tallián Gyula Street 20-32, Kaposvár, 7400 Hungary; 2 Department of Anaesthesiology and Intensive Therapy, Faculty of Medicine, University of Szeged, Semmelweis Street 6, Szeged, 6725 Hungary; 3 Department of Radiology, Teaching Hospital Mór Kaposi, Tallián Gyula Street 20-32, Kaposvár, 7400 Hungary; 4 Department of Cardiology, Teaching Hospital Mór Kaposi, Tallián Gyula Street 20-32, Kaposvár, 7400 Hungary; 5 Department of Hematology, Teaching Hospital Mór Kaposi, Tallián Gyula Street 20-32, Kaposvár, 7400 Hungary; 6 Faculty of Health Sciences, Doctoral School, University of Pécs, Vörösmarty Mihály Street 4, Pécs, 7621 Hungary; 7 Institute of Emergency Care and Pedagogy of Health, Faculty of Health Sciences, University of Pécs, Vörösmarty Mihály Street 4, Pécs, 7621 Hungary

**Keywords:** Differential diagnosis, Diffuse large B cell lymphoma, Cardiac metastasis, Coronary artery, Acute coronary syndrome

## Abstract

**Background:**

Cardiac tumors are very uncommon compared to other cardiac diseases. Their clinical symptoms can vary from absent to non-specific. The most common symptoms are arrhythmias, blood flow obstruction due to valvular dysfunction, shortness of breath, systemic embolization, and accumulation of pericardial fluid. Hereby, we describe a very rare case of a diffuse large B cell lymphoma patient who presented with the symptoms and signs of acute coronary syndrome (ACS) but the patient’s complaints were caused by his intramyocardial lymphoma metastasis.

**Case presentation:**

Forty-eight-year-old diffuse large B cell lymphoma patient was admitted to our emergency department with chest pain, effort dyspnea, and fever. The patient had normal blood pressure, blood oxygen saturation, sinus tachycardia, fever, crackles over the left lower lobe, novum incomplete right bundle branch block with Q waves and minor ST alterations, elevated C-reactive protein, high-sensitivity troponin-T, and d-dimer levels. Chest X-ray revealed consolidation on the left side and enlarged heart. Bed side transthoracic echocardiography showed inferior akinesis with pericardial fluid. Coronary angiography showed no occlusion or significant stenosis. Chest computed tomography demonstrated the progression of his lymphoma in the myocardium. He was admitted to the Department of Hematology for immediate chemotherapy and he reached complete metabolic remission, followed by allogeneic hematopoietic stem cell transplantation. Unfortunately, about 9 months later, he developed bone marrow deficiency consequently severe sepsis, septic shock, and multiple organ failure what he did not survive.

**Conclusions:**

Our case demonstrates a very rare manifestation of a heart metastasis. ACS is an unusual symptom of cardiac tumors. But our patient’s intramyocardial lymphoma in the right atrium and ventricle externally compressed the right coronary artery and damaged the heart tissue, causing the patient’s symptoms which imitated ACS. Fortunately, the quick diagnostics and immediate aggressive chemotherapy provided the patient’s remission and suitability to further treatment.

## Background

Heart tumors are rare findings. The frequency of primary cardiac tumors ranges between 0.001 and 0.3% [1–3]. Seventy-five percent of them are benign and 25% are malignant [4]. Atrial myxoma is the most frequent primary tumor in adults, and rhabdomyosarcoma is the most prevalent in children [5]. No malignant tumors are known which diffuse preferentially to the heart, some do involve the heart more often than others, for example melanoma and primary mediastinal tumors. The incidence of cardiac metastases reported in literature is highly variable, ranging from 2.3 to 18.3% in tumor patients. At autopsy, they are more frequently found than the primary neoplasms of the heart [4–7]. The most common cardiac metastases are carcinomas, tissue sarcomas, melanomas, and lymphomas [8]. Most secondary cardiac tumors are clinically silent (90%) and are often diagnosed postmortem [9].

The incidence of emergency department visits among oncology patients ranges from 1 to 12% up to 30% according to reviews [10–13]. Estimated frequency of hematological malignancies in male patients is around 7.5% [14]. Diffuse large B cell lymphoma (DLBCL) is the most common type of non-Hodgkin lymphoma (NHL) [15], representing approximately 24% of NHL patients [16]. These statistical data suggest that the occurrence of patients with hematological malignancies at the emergency department is around 0.5% or lower, which is in accordance with the statistics of our Department of Emergency. Approximately 8-15% of all lymphoma patients have cardiac involvement [17, 18]. In the few existing cases in the medical literature, the heart seems to be more often involved in non-Hodgkin’s lymphoma, whereas the pericardium is more often metastasized by Hodgkin’s lymphoma [19]. These data show that the incidence of cardiac lymphoma in emergency departments is very uncommon.

Clinical manifestations of the heart tumors can be various and non-specific which often leads to their delayed diagnosis and management. The most commonly reported symptoms include valvular dysfunctions, arrhythmias, accumulation of pericardial fluid with or without tamponade, and peripheral embolization with certain deficits. But some tumors do not cause any symptoms and remain only incidental findings [20, 21]. Secondary cardiac lymphoma patients also have a very poor outcome due to the high risk of sudden death. Most patients die before the start of therapy. For this reason, cardiac involvement should be diagnosed early [22].

## Case presentation

The 48-year-old male patient was diagnosed with germinal center (GC) subgroup of DLBCL developed from follicular lymphoma in 2017, after having a 6 months history of submandibular and inguinal lymphadenomegaly, and hepatomegaly.

He was in stage IV according to Ann Arbor classification at the time of diagnosis, and his international prognostic index was 3.

He received several regimes of chemotherapy but has not reached complete remission. Finally, a high dose methotrexate and rituximab treatment has resulted in significant morpho-metabolic regression in the lymphoma manifestations. Therefore, homologous hematopoietic stem cell transplantation (HSCT) was planned. In July 2019, the patient underwent an unsuccessful attempt to collect enough stem cells for HSCT. Two weeks later, he was admitted to our Department of Emergency with complaints of effort dyspnea, chest pain, and fever. He received level 3 (urgent) on the triage scale (the Hungarian Emergency Triage System was adapted from the Canadian Triage Acuity Scale).

Differential diagnosis of chest pain includes benign and life threatening conditions, which require rapid identification and treatment. These life threatening diseases are not limited to but include acute coronary syndrome, pulmonary embolism, tension pneumothorax, aortic dissection, esophageal rupture, and pericarditis/pericardial tamponade. Physical examination and basic findings can narrow the list of possible diagnoses [23, 24].

**Fig. 1:**
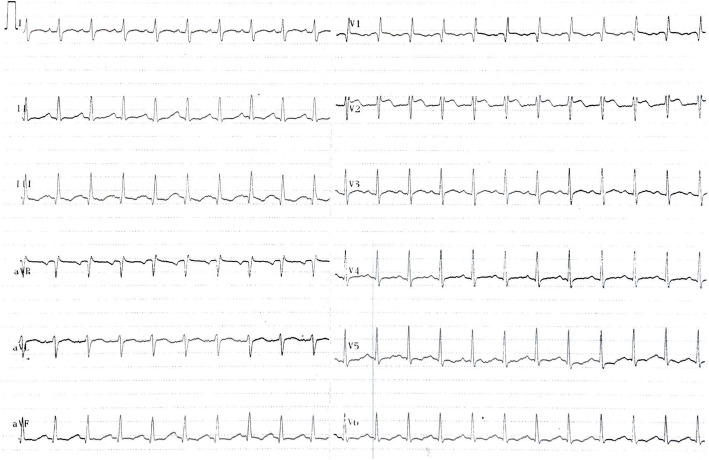
Electrocardiogram of the patient. Electrocardiogram of the patient shows sinus tachycardia, incomplete right bundle branch block with Q waves, and minor ST elevations in leads II, III, and aVF

On physical examination, the patient had normal blood pressure (138/80 mmHg) and blood oxygen saturation (96%), sinus tachycardia (133 beats per minute), and body temperature of 38.9 °C. On auscultation of the lungs, mild crackles were heard over the left lower lobe. The patient’s electrocardiogram (ECG) revealed sinus tachycardia, and novum incomplete right bundle branch block with Q waves and minor ST elevations in leads II, III and aVF (Fig. 1).

His ECG findings (ST elevation, right bundle brunch block) raised the likeliness of ACS and pulmonary embolism [25–27]. Tension pneumothorax and cardiac tamponade were much less probable because the patient’s blood oxygen saturation, and blood pressure were normal, had no respiratory distress, his neck veins were not distended. Heart sounds were well audible, and ECG showed no low voltage. Auscultation and percussion of the lungs showed no unilateral difference [28, 29]. Based on the characteristics of the chest pain, aortic dissection was improbable. Its improbability was further supported by the presence of normal blood pressure and pulse on both arms, and the lack of other additional findings, like abdominal pain, migrating pain, syncope, and neurologic deficits [30]. Esophageal rupture is most common in patients who have underwent instrumental examination of the esophagus or have had heavy vomiting [31]. The patient had none of these. The presence of fever and the fact that the patient is immunocompromized strengthened feasibility of pneumonia. Crackles over the left lower lobe of the lungs also fortified this. The complaints and basic physical findings primarily directed us towards acute coronary syndrome, pulmonary embolism, pneumonia, but further laboratory examinations and imaging were needed to the exact diagnosis.

From his laboratory findings at presentation elevated C-reactive protein (42.3 mg/l), high-sensitivity troponin-T (hsTnT) (432 pg/ml), and d-dimer (6376 ug/l) levels were notable (Table 1). After further questioning, it was also revealed that the patient forgot to administer his prophylactic low molecular weight heparin (LMWH) the day before his admission, which further supported the possibility of thromboembolism.

**Table 1:** Laboratory parameters of the patient

Laboratory parameter	Reference range	Test results				
		Day 1	Day 3	Day 4	Day 9	Day 11
white blood cells (G/L)	4.5–10.1	5.25	4.48	3.88		1.21
red blood cells (T/L)	4.1–5.1	2.51	2.45	2.29		2.88
hemoglobin (g/L)	140–175	87	81	76		95
hematocrite (%)	40–52	25.8				
platelate count (G/L)	100–450	105				
INR	0.8–1.2	1.06				
D-dimer (μg/L)	0–500	**6376**	**3514**			
CRP (mg/L)	0–5	**42.3**	**89.7**		**12.6**	
glucose (mmol/L)	3.3–5.5	5.1				
sodium (mmol/L)	132–146	136				
potassium (mmol/L)	3.3–5.4	4.07				
AST (U/L)	0–45	23				
ALT (U/L)	0–50	12				
GGT (U/L)	8–60	44				
ALP (U/L)	40–130					
Amilase (U/L)	28–100					
CK (U/L)	20–200	179				
LDH (U/L)	240–280	**808**	**712**		420	
se/bilirubin (μmol/L)	0–20	12				
GFR (ml/min)	90–1000	66	72			
creatinine (μmol/L)	62–106	**114**	105		91	
urea (mmol/L)	0.00–11.9	4.4				
hsTnT (pg/ml)	0–14	**432.1**	**329.6**			**103**

Laboratory parameters of the patient at the Department of Emergency (day 1) and at the Department of Hematology (days 2–11) (numbers in bold indicate alterations from the normal value)

**Fig. 2:**
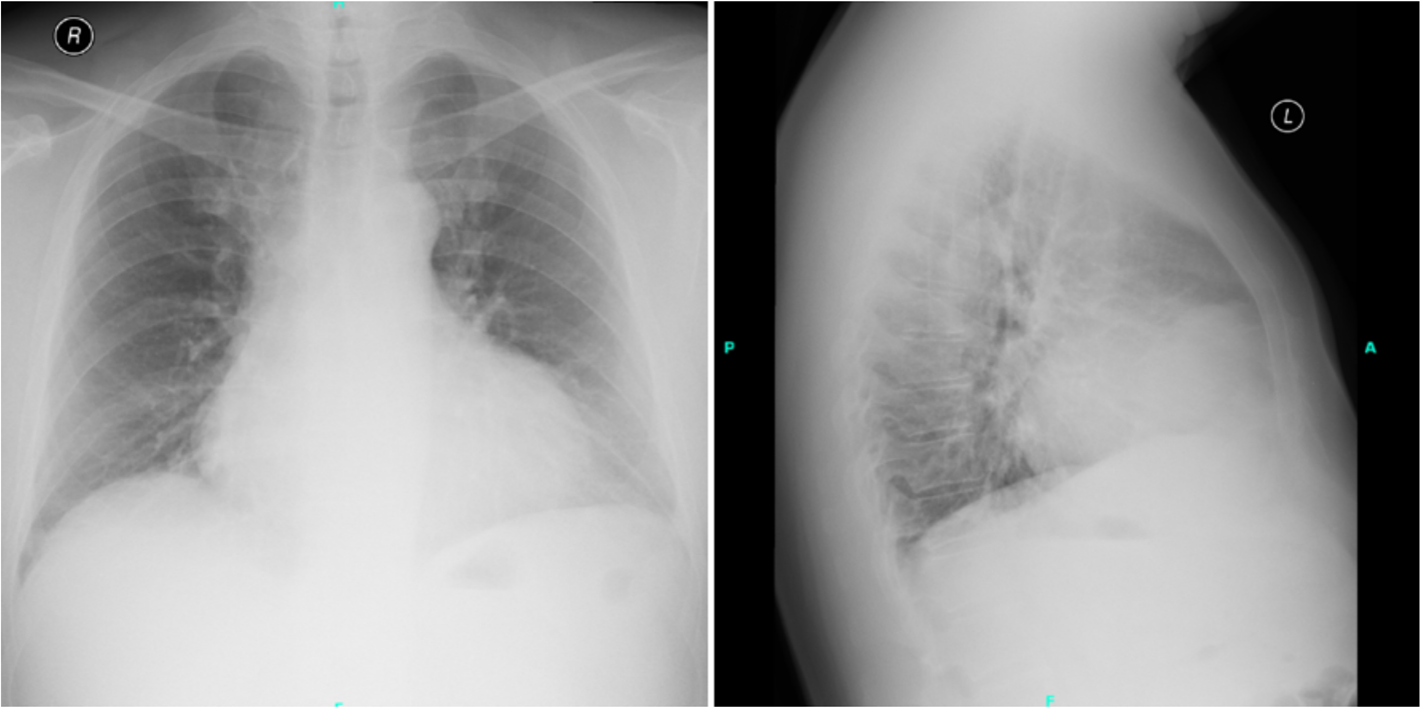
Chest X-ray image of the patient. Chest X-ray demonstrated small amount of pleural fluid in the left lateral sinus, 6.5 × 4.5 cm large consolidation or dystelectasis on the left side close to the significantly enlarged heart

Chest X-ray (Fig. 2) showed consolidation or dystelectasis on the left side and significantly enlarged heart compared to the previous images. The enlarged heart raised the possibility of presence of pericardial fluid.

Because of the probability of pericardial effusion bed side transthoracic echocardiography (TTE) was performed which depicted inferior akinesis, reduced left ventricular function with 4–6 mm pericardial fluid and suggested coronarography following chest computed tomography (CT) to rule out pulmonary embolism.

Quick assessment of the chest CT images demonstrated no significant embolism.

**Fig. 3:**
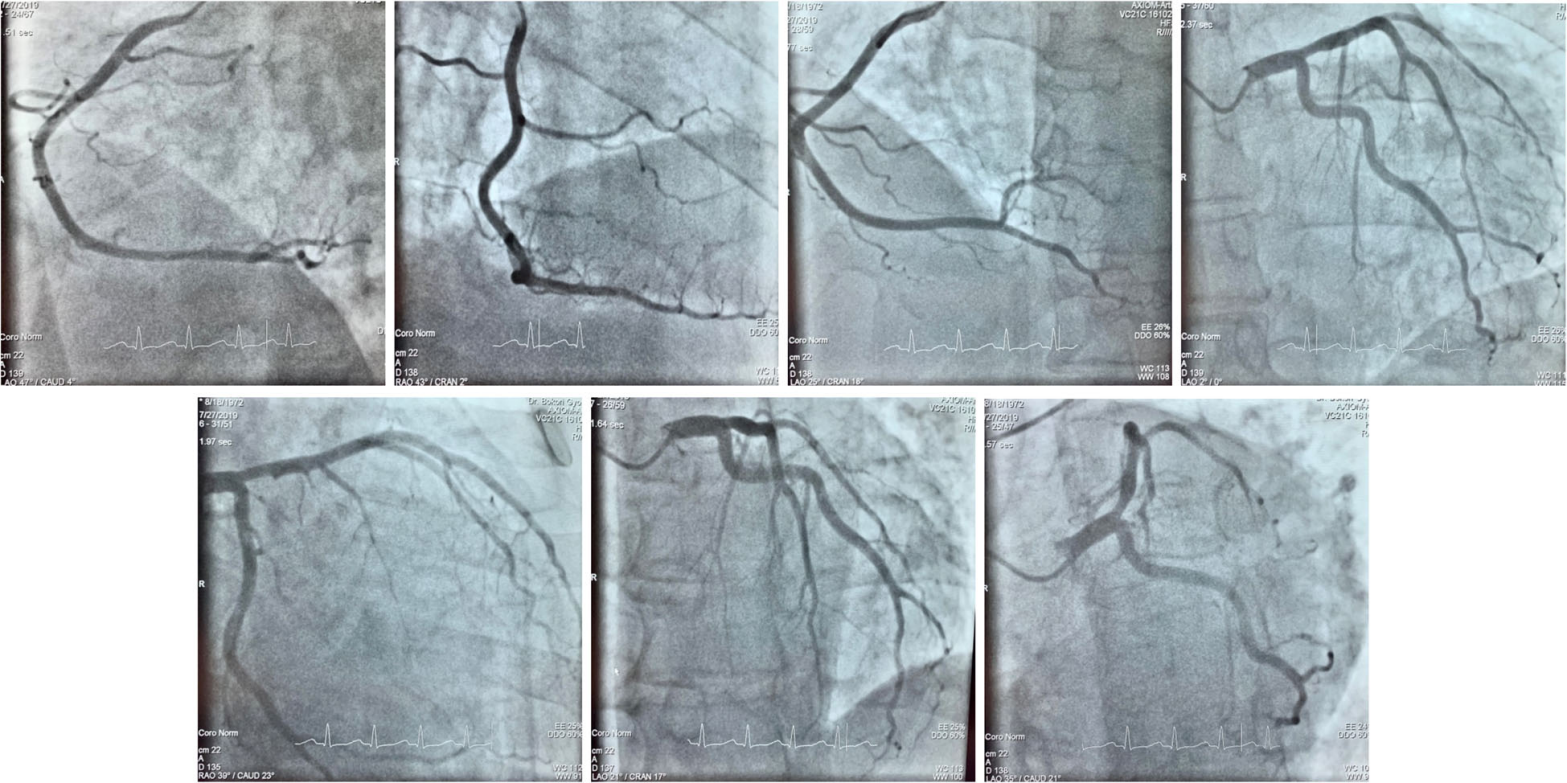
Coronary angiography images of the patient. Coronary angiography showed no occlusion or significant stenosis in the coronary arteries

Coronary angiography was immediately performed showed no occlusion or significant stenosis (Fig. 3).

**Fig. 4:**
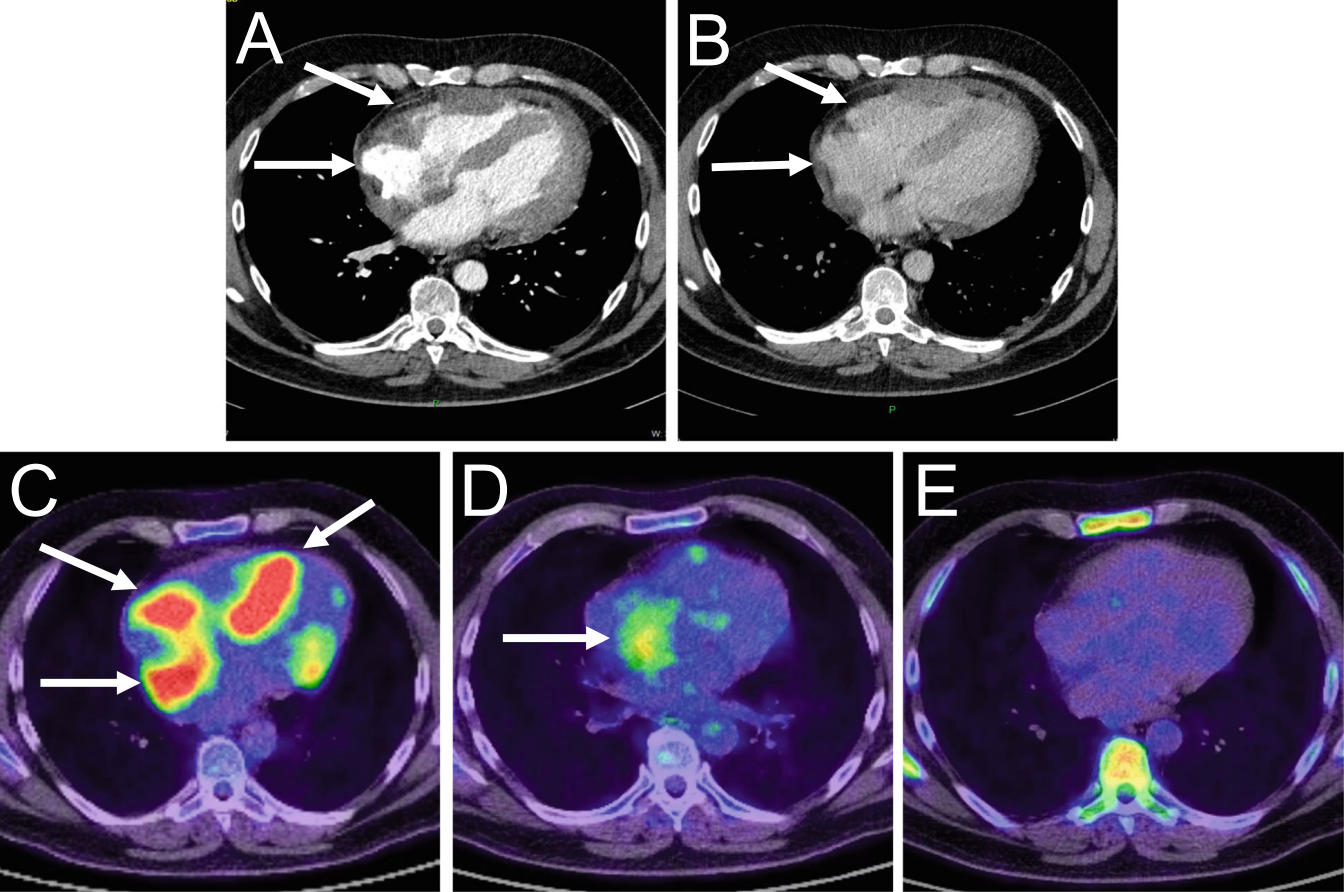
Chest CT and PET/CT images of the patient. **a**, **b** Contrast-enhanced chest CT transaxial reconstruction shows the inhomogeneous hypodense tissue in the right atrium and ventricle and circumferential pericardial effusion (arrows). **c**–**e** Fused 18-fluorodeoxyglucose (FDG) PET/CT image axial sections demonstrate the right atrium and ventricle mass along with the FDG uptake within the tumor (arrows), the images were made before (**c**), during (**d**), and after (**e**) the patient received chemotherapy to treat his intramyocardial metastasis

In-depth analysis of the patient’s chest CT images (Fig. 4) revealed abnormal inhomogeneous hypodense tissue in the walls of the right ventricle and atrium considered as progression of his lymphoma. The size of this lesion was 50 × 22 mm in the lateral wall of the right ventricle and 84 × 59 mm in the wall of the right atrium, which externally compressed the right coronary artery. The CT images excluded embolism but supported the result of the chest X-ray demonstrating pneumonia.

The patient’s complaints were caused by the progression of his lymphoma; therefore, he was admitted to the Department of Hematology for further treatment. The patient received immediate therapy according to R-GemOx (gemcitabine-oxaliplatin plus rituximab) protocol complemented with venetoclax then he underwent polatuzumab-vedotin and rituximab, bendamustin treatment and his symptoms ameliorated, he reached complete metabolic remission. He went through allogeneic HSCT. Unfortunately, 9 months later, he was again admitted to our hospital with severe dyspnea and chest pain due to bone marrow deficiency. He developed severe sepsis, then septic shock with multiple organ failure what he did not survive.

## Discussion and conclusions

The WHO classification of tumors of hematopoietic and lymphoid tissues divides DLBCL into several subtypes including GC B cell subgroup [32]. DLBCL is the most frequent lymphoma subtype, accounts for about one-third of the newly diagnosed lymphoma cases worldwide [33]. The different subtypes show significant differences in pathogenesis and clinical manifestation and most importantly are treated differently from classical DLBCL. Therefore, the exact origin of the malignant cell and its molecular background must be determined [32].

Cardiac metastases are rare, and usually develop clinically silent or have non-specific symptoms. Therefore, they are difficult to diagnose and are frequently detected postmortem during an autopsy. In patients with lymphoma, the cardiac involvement is seen in 8–15% of the cases and most of them are of B cell origin [34]. Clinical manifestations are highly variable, including heart failure, arrhythmias, valvular disease, and cardiac tamponade [35]. Due to the varying clinical presentations of cardiac lymphoma metastases, the detection is often incidental.

If the signs and symptoms of cardiac disease are present, non-invasive imaging is key in prompt diagnosis [36, 37]. Chest radiography may indicate cardiomegaly caused by pericardial effusion, abnormal shape of the heart, or lung congestion [8]. Echocardiography is the most frequently used non-invasive imaging technique to assess the heart. TTE is useful in the initial evaluation of suspected cardiac disease, but sometimes transesophageal echocardiography is required for a more accurate assessment [38]. The diagnostic accuracy of echocardiography is around 80% in the evaluation of suspected cardiac tumors [39]. CT and magnetic resonance imaging provide additional detail of the surrounding extracardiac structures, including the lungs, mediastinum, and pleura [40]. Positron emission tomography (PET)/CT may provide additional utility and improved accuracy in earlier detection of metastases involving the heart [41].

There is no standard treatment of cardiac metastases in lymphomas. The therapy of choice is mostly influenced by the location of the metastasis and the patient’s condition. The individualized treatment often means interdisciplinary cooperation of oncologists, radiotherapists, and surgeons [3].

In our case, the patient showed non-specific symptoms, such as effort dyspnea, chest discomfort, and even fever. His physical status, laboratory, and ECG findings supported the presence of more common conditions. Fever and crackles found on physical examination, the 2-week-old history of chest pain and the fact that the patient is actively treated with chemotherapy due to hematological malignancy suggested pneumonia in the background. His ECG finding with the previously non-described incomplete right bundle branch block not only strengthened the possibility of pneumonia but also raised the possibility of pulmonary embolism and acute coronary syndrome. Therefore, the patient’s blood was tested for infection, d-dimer, and high-sensitivity troponin-T, and chest X-ray was performed. The first result confirmed pneumonia but the high d-dimer and hsTnT indicated the need of further investigation. High d-dimer suggested the possibility of pulmonary embolism. He even admitted forgetting to administer his prophylactic LMWH the day before his admission.

Pulmonary embolism can be accompanied by elevated hsTnT level [42] and vice versa elevated d-dimer level may occur in ischemic heart disease [43]. The GRACE score of the patient was 101 points, his hsTnT level was high enough to fulfill a high-risk criterium for acute coronary syndrome. His Well’s score was 5.5 suggesting a moderate risk for pulmonary embolism [43]. The possibility of both conditions, myocardial infarction and pulmonary embolism, was present consequently; thus, one of them must have to be excluded. We performed TTE to detect the presence of right ventricular dilatation as a sign for acute pulmonary embolism or myocardial hypo-akinesis due to myocardial infraction. TTE showed inferior akinesis; still, the possibility of pulmonary embolism was not clearly excluded. According to the guideline of the European Society of Cardiology if the patient’s initial evaluation suggests pulmonary embolism CT angiography is recommended before coronary angiography [42]. Chest CT can effectively exclude not only pulmonary embolism, but other causes of chest pain as well which, if untreated, can be associated with high mortality. Quick assessment of the chest CT showed no embolism. Thus, the coronary angiography was immediately performed in correlation with the early invasive strategy [42], but no occlusion or significant stenosis was found in the coronary arteries. While the coronary angiography was done, the fine analysis of the chest CT revealed the intramyocardial progression of the patient’s lymphoma.

His fever was caused by concomitant consolidation in the left lower lobe and was successfully cured by imipenem-cilastatin combination. High d-dimer was most likely the consequence of the patient’s lymphoma and pneumonia and not the result of thromboembolism as we first predicted [44]. The high troponin-T level was observed because the lymphoma damaged the heart tissue leading to the release of this protein [42].

Most of his findings suggested thromboembolism, myocardial infarction, or pneumonia in the background of his complaints rather than the presence of intramyocardial lymphoma.

This case illustrate that rarely the symptoms of ACS and high troponin-T levels can be observed without occluded or stenotic coronary arteries caused by direct myocardial damage due to the intramyocardial progression of a malignant lymphoid tissue and its external compression on coronary arteries leading to inappropriate blood supply of the heart. The patient was fortunate because of the early diagnosis (Table 2) and successful treatment of his cardiac lymphoma metastasis provided him almost a year survival.

**Table 2:** Time points of diagnostic procedures and the total time until the final diagnosis of the patient

Time (min)	Test
0	Physical examination
32	Chest X-ray
47	Laboratory test
250	Echocardiography
311	Chest computed tomography
336	Coronarography
386	Department of Hematology
Total: 6 h 26 min	

All these diagnostic procedures were necessary to the final diagnosis because all the excluded diseases would have needed different therapy. If the patient had ACS thrombocyte aggregation inhibition, stent implantation would have been the treatment. In the case of pulmonary embolism, he would have received anticoagulation. But none of these therapies would have actually cured his problem and would have leaded to the death of the patient very rapidly. Indicating immediate chemotherapy as an adequate remedy for an emergency department patient’s condition is scarcity, but as our case exemplifies even this should never be excluded.

The patient was stable and his vital parameters were satisfactory without any support; therefore, his disposition to intensive care unit was not necessary. However, cardiac tumors often cause arrhythmias, valvular dysfunction, embolization, and accumulation of pericardial fluid. These can be life threatening; their earliest recognition is key to appropriate interventions. Thus, the patient must have been tightly monitored at a sub-intensive care unit.

To conclude, primary and secondary cardiac tumors are uncommon diseases. Their clinical symptoms are variable, and acute coronary syndrome is among the rarest. Our patient’s case is unique because he presented the symptoms of more common conditions, which affect large populations, such as pulmonary embolism, pneumonia, ACS, aortic dissection, pneumothorax, rupture of the esophagus or pericarditis. The early diagnosis and management of these diseases in emergency department settings is crucial for the survival of the patient. But the patient’s findings revealed cardiac lymphoma metastasis which externally compressed the right coronary artery, leading to symptoms mimicking ACS. Fortunately, the quick diagnosis and efficient cooperation with cardiologists, radiologists, and hematologists permitted the instant adequate therapy to the patient.

## Data Availability

The datasets used and/or analyzed during the current study are available from the corresponding author on reasonable request.

## Abbreviations

ACS: Acute coronary syndrome
CT: Computed tomography
DLBCL: Diffuse large B cell lymphoma
ECG: Electrocardiogram
GC: Germinal center
HSCT: Hemopoietic stem cell transplantation
LMWH: Low molecular weight heparin
NHL: Non-Hodgkin lymphoma
TTE: Transthoracic echocardiography

## References

[1] Centofanti P, Di Rosa E, Deorsola L, Actis Dato GM, Patanè F, La Torre M, et al. Primary cardiac tumors: Early and late results of surgical treatment in 91 patients. Ann Thorac Surg. 1999;68:1236–41. 10.1016/S0003-4975(99)00700-6. 10.1016/s0003-4975(99)00700-6 10543485

[2] Patel J, Sheppard MN (2010). Pathological study of primary cardiac and pericardial tumours in a specialist UK Centre: Surgical and autopsy series. Cardiovasc Pathol.

[3] Hoffmeier A, Sindermann JR, Scheld HH, Martens S (2014). Herztumoren - Diagnostik und chirurgische therapie. Dtsch Arzteblatt Int.

[4] Amano J, Nakayama J, Yoshimura Y, Ikeda U (2013). Clinical classification of cardiovascular tumors and tumor-like lesions, and its incidences. Gen Thorac Cardiovasc Surg.

[5] Reynen K (1996). Frequency of primary tumors of the heart. Am J Cardiol.

[6] Bussani R, De-Giorgio F, Abbate A, Silvestri F (2007). Cardiac metastases. J Clin Pathol.

[7] Al-Mamgani A, Baartman L, Baaijens M, De Pree I, Incrocci L, Levendag PC (2008). Cardiac metastases. Int J Clin Oncol.

[8] Strecker T, Rösch J, Weyand M, Agaimy A (2012). Primary and metastatic cardiac tumors: Imaging characteristics, surgical treatment, and histopathological spectrum: a 10-year-experience at a German heart center. Cardiovasc Pathol.

[9] Burazor I, Aviel-Ronen S, Imazio M, Goitein O, Perelman M, Shelestovich N, Radovanovic N, Kanjuh V, Barshack I, Adler Y (2018). Metastatic cardiac tumors: From clinical presentation through diagnosis to treatment. BMC Cancer.

[10] Lash RS, Bell JF, Reed SC, Poghosyan H, Rodgers J, Kim KK, Bold RJ, Joseph JG (2017). A systematic review of emergency department use among cancer patients. Cancer Nurs.

[11] Mayer DK, Travers D, Wyss A, Leak A, Waller A (2011). Why do patients with cancer visit emergency departments? Results of a 2008 population study in North Carolina. J Clin Oncol.

[12] Lash RS, Bell JF, Bold RJ, Joseph JG, Cress RD, Wun T, Brunson A, Romano P (2017). Emergency department use by recently diagnosed cancer patients in California. J Community Support Oncol.

[13] Rivera DR, Gallicchio L, Brown J, Liu B, Kyriacou DN, Shelburne N (2017). Trends in adult cancer–related emergency department utilization: An analysis of data from the nationwide emergency department sample. JAMA Oncol.

[14] Roman E, Smith AG (2011). Epidemiology of lymphomas. Histopathology..

[15] Taylor J, Xiao W, Abdel-Wahab O (2017). Diagnosis and classification of hematologic malignancies on the basis of genetics. Blood..

[16] Liu Y, Barta SK (2019). Diffuse large B-cell lymphoma: 2019 update on diagnosis, risk stratification, and treatment. Am J Hematol.

[17] Silvestri F, Bussani R, Pavletic N, Mannone T (1997). Metastases of the heart and pericardium. G Ital Cardiol.

[18] Chinen K, Izumo T (2005). Cardiac involvement by malignant lymphoma: A clinicopathologic study of 25 autopsy cases based on the WHO classification. Ann Hematol.

[19] Moore JA, DeRan BP, Minor R, Julie A, Fraker TD (1992). Transesophageal echocardiographic evaluation of intracardiac lymphoma. Am Heart J.

[20] Bossert T (2005). Surgical experience with 77 primary cardiac tumors. Interact Cardiovasc Thorac Surg.

[21] Schrepfer S, Deuse T, Detter C, Treede H, Koops A, Boehm DH, Willems S, Lacour-Gayet F, Reichenspurner H (2003). Successful resection of a symptomatic right ventricular lipoma. Ann Thorac Surg.

[22] Tanaka T, Sato T, Akifuji Y, Sakamoto M, Shio H, Ueki J (1996). Aggressive non-Hodgkin’s lymphoma with massive involvement of the right ventricle. Intern Med.

[23] Fass R, Achem SR (2011). Noncardiac chest pain: Epidemiology, natural course and pathogenesis. J Neurogastroenterol Motil.

[24] Ebell MH (2011). Evaluation of chest pain in primary care patients. Am Fam Physician.

[25] Fanaroff AC, Rymer JA, Goldstein SA, Simel DL, Newby LK (2015). Does this patient with chest pain have acute coronary syndrome?: The rational clinical examination systematic review. JAMA.

[26] Miranda DF, Lobo AS, Walsh B, Sandoval Y, Smith SW (2018). New Insights Into the Use of the 12-Lead Electrocardiogram for Diagnosing Acute Myocardial Infarction in the Emergency Department. Can J Cardiol.

[27] Stein PD, Henry JW (1997). Clinical characteristics of patients with acute pulmonary embolism stratified according to their presenting syndromes. Chest..

[28] Roberts DJ, Leigh-Smith S, Faris PD, Blackmore C, Ball CG, Robertson HL, Dixon E, James MT, Kirkpatrick AW, Kortbeek JB, Stelfox HT (2015). Clinical presentation of patients with tension pneumothorax: A systematic review. Ann Surg.

[29] Vakamudi S, Ho N, Cremer PC (2017). Pericardial Effusions: Causes, Diagnosis, and Management. Prog Cardiovasc Dis.

[30] Strayer RJ (2017). Thoracic Aortic Syndromes. Emerg Med Clin North Am.

[31] Kang SG, Song HY, Lim MK, Yoon HK, Goo DE, Sung KB (1998). Esophageal rupture during balloon dilation of strictures of benign or malignant causes: prevalence and clinical importance. Radiology..

[32] Choi SM, O’Malley DP (2018). Diagnostically relevant updates to the 2017 WHO classification of lymphoid neoplasms. Ann Diagn Pathol.

[33] Smith A, Crouch S, Lax S, Li J, Painter D, Howell D, Patmore R, Jack A, Roman E (2015). Lymphoma incidence, survival and prevalence 2004-2014: Sub-type analyses from the UK’s Haematological Malignancy Research Network. Br J Cancer.

[34] Fujita Y, Ikebuchi M, Tarui S, Irie H (2009). Successful combined treatment of primary cardiac malignant lymphoma with urgent cardiac operation and chemotherapy. Circ J.

[35] Kim JK, Sindhu K, Bakst RL (2019). Cardiac metastasis in a patient with head and neck cancer: a case report and review of the literature. Case Rep Otolaryngol.

[36] Grebenc ML, Rosado De Christenson ML, Burke AP, Green CE, Galvin JR (2000). Primary cardiac and pericardial neoplasms: Radiologic-pathologic correlation. Radiographics..

[37] Jellis C, Hunter A, Sutton R (2009). Multimodal imaging of an atrial myxoma. Cardiovasc Pathol.

[38] Alam M, Rosman HS, Grullon C (1995). Transesophageal echocardiography in evaluation of atrial masses. Angiology..

[39] Nomoto N, Tani T, Konda T, Kim K, Kitai T, Ota M, Kaji S, Imai Y, Okada Y, Furukawa Y (2017). Primary and metastatic cardiac tumors: Echocardiographic diagnosis, treatment and prognosis in a 15-years single center study. J Cardiothorac Surg.

[40] Chiles C, Woodard PK, Gutierrez FR, Link KM (2001). Metastatic involvement of the heart and pericardium: CT and MR imaging. Radiographics..

[41] Mittal B, Manohar K, Kashyap R, Bhattacharya A, Varma S, Agrawal K (2012). FDG PET/CT in detection of metastatic involvement of heart and treatment monitoring in non-Hodgkin′s lymphoma. World J Nucl Med.

[42] Roffi M, Patrono C, Collet JP, Mueller C, Valgimigli M, Andreotti F, et al. 2015 ESC Guidelines for the management of acute coronary syndromes in patients presenting without persistent st-segment elevation: Task force for the management of acute coronary syndromes in patients presenting without persistent ST-segment elevation of . European Heart Journal. 2016;37:267–315. 10.1093/eurheartj/ehv320. 10.1093/eurheartj/ehv320 26320110

[43] Konstantinides SV, Meyer G, Becattini C, Bueno H, Geersing G-J, Harjola V-P, Huisman MV, Humbert M, Jennings CS, Jiménez D, Kucher N, Lang IM, Lankeit M, Lorusso R, Mazzolai L, Meneveau N, Áinle FN, Prandoni P, Pruszczyk P, Righini M, Torbicki A, van Belle E, Zamorano JL, The Task Force for the diagnosis and management of acute pulmonary embolism of the European Society of Cardiology (ESC) (2019). 2019 ESC Guidelines for the diagnosis and management of acute pulmonary embolism developed in collaboration with the European Respiratory Society (ERS). Eur Respir J.

[44] Kabrhel C, Mark Courtney D, Camargo CA, Plewa MC, Nordenholz KE, Moore CL (2010). Factors associated with positive d-dimer results in patients evaluated for pulmonary embolism. Acad Emerg Med.

